# Multifunctional Prussian-Blue-Based Hydrogel for Photothermal Antibacterial and Infected Wound Regeneration

**DOI:** 10.3390/polym18141688

**Published:** 2026-07-09

**Authors:** Shiqi Gao, Minzhen Liu, Jiteng Sun, Zhicheng Su, Ziyun Liao, Peiyu Li, Yunqi Jiang, Can Fu, Guangyu Pan

**Affiliations:** 1 Guangxi Key Laboratory of Diabetic Systems Medicine, Guilin Medical University, Guilin 541199, China; shiqi@stu.glmc.edu.cn (S.G.); 19175931257@163.com (M.L.); 13978423864@stu.glmc.edu.cn (Z.S.); perry1271522559@gmail.com (P.L.); 2 School of Pharmacy, Guilin Medical University, Guilin 541199, China; 3 School of Artificial Intelligent Medicine, Guilin Medical University, Guilin 541199, China; 4 School of Basic Medical Sciences, Guilin Medical University, Guilin 541199, China; 5 Key Laboratory of Biochemistry and Molecular Biology (Guilin Medical University), Education Department of Guangxi Zhuang Autonomous Region, Guilin 541199, China

**Keywords:** wound dressing, prussian blue, photothermal sterilization, hydrogel, infected wound

## Abstract

To address the challenges associated with prolonged inflammatory phases and delayed healing in clinically infected wounds, this research developed a multifunctional PB@GC@OD hydrogel integrating self-healing properties, injectability, and photothermal antibacterial efficacy. The hydrogel was constructed using oxidized dextran (OD) and glycol chitosan (GC) as the matrix, which were dynamically cross-linked via a Schiff-base reaction to form the GC@OD hydrogel. Subsequently, the photothermal agent prussian blue (PB) was incorporated to fabricate the PB@GC@OD hydrogel. The resulting PB@GC@OD hydrogel demonstrated robust self-healing capabilities and excellent injectability. Upon exposure to 808 nm near-infrared (NIR) irradiation, the hydrogel achieved efficient photothermal conversion, rapidly inducing localized hyperthermia that effectively eliminated *Staphylococcus aureus*, *Escherichia coli*, and methicillin-resistant *Staphylococcus aureus* (MRSA). In a mouse model of MRSA-infected wounds, the hydrogel not only maintained a moist wound microenvironment but also eradicated pathogenic bacteria via photothermal therapy, thereby significantly accelerating the healing process. Moreover, the hydrogel demonstrated favorable biocompatibility and long-term safety. Therefore, the PB@GC@OD hydrogel integrates photothermal sterilization, self-healing, injectability, hemostasis, and biocompatibility into a single platform, presenting a promising strategy for synergistic therapy and tissue regeneration in bacterially infected wounds.

## 1. Introduction

The skin, the largest organ of the human body, serves as a crucial physiological barrier against external physical damage, chemical stimuli, and microbial invasion [1,2]. Traumatic injury can compromise skin integrity, leading to wound formation. Under normal physiological conditions, skin wounds typically heal via a self-regulatory mechanism, a highly coordinated biological process encompassing four overlapping yet sequential phases: hemostasis, inflammation, proliferation, and remodeling [3,4,5,6,7]. Among these, the inflammatory phase is pivotal, governing the progression of the entire healing process [8,9,10,11]. However, open wounds remain highly vulnerable to bacterial colonization during clinical management and care [12,13]. Wound infection can prolong the inflammatory phase, thereby delaying the entire healing process [14]. Consequently, the timely eradication of infection and the resolution of inflammation are critical prerequisites for effective wound repair [15,16,17,18].

In clinical wound management, wound dressings are utilized as a cornerstone of therapeutic intervention. They provide a physical barrier, maintain a moist environment optimal for healing, and facilitate tissue regeneration [19,20]. Hydrogel dressings, in particular, have garnered considerable interest due to their unique physicochemical properties, excellent biocompatibility, tunable mechanical performance, and extracellular matrix-like structure. They not only promote cell migration and proliferation but also effectively mitigate wound desiccation, thereby providing a conducive microenvironment for wound repair [21,22,23].

Among various hydrogel matrix materials, dextran, a biocompatible and non-cytotoxic natural polysaccharide, has emerged as a promising candidate material for functional hydrogels owing to its excellent biocompatibility, ease of modification, and bioactivity [24,25,26]. The vicinal diols on the dextran backbone can be oxidized by sodium periodate to yield pendant aldehyde functionalities, which can subsequently form hydrogels via dynamic covalent imine bond cross-linking with amine-rich polysaccharides, such as chitosan [21]. Glycol Chitosan (GC), a water-soluble chitosan derivative, features abundant amine groups alongside excellent biocompatibility and biodegradability, rendering it an ideal candidate for hydrogel fabrication [27,28,29,30].

The incorporation of antimicrobial agents into hydrogels constitutes a promising therapeutic strategy for treating infected wounds. Among various antimicrobial approaches, photothermal antibacterial therapy utilizes photothermal agents to convert near-infrared (NIR) light energy into heat, effectuating bacterial ablation through localized hyperthermia. This method physically disrupts bacterial structures, offering advantages such as high efficiency and a low propensity for inducing drug resistance, which is particularly significant in addressing antibiotic resistance issues [31,32,33,34,35,36]. Prussian Blue (PB), an inorganic coordination polymer with a distinctive lattice structure, has emerged as a premier next-generation photothermal agent owing to its intense NIR absorption, superior photothermal conversion efficiency, and excellent biocompatibility profile [37,38,39,40,41,42,43].

**Figure 1:**
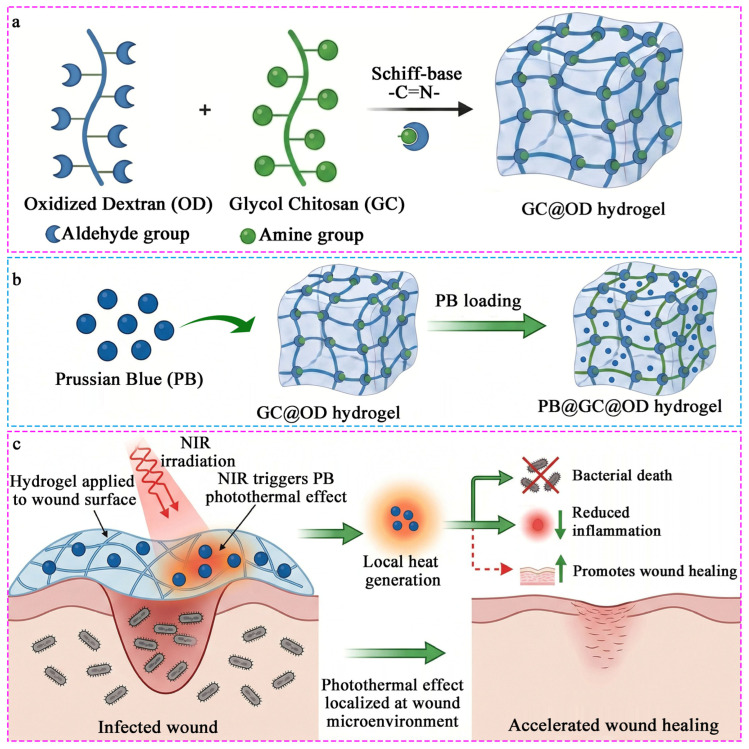
Fabrication of GC@OD hydrogel (**a**) and PB@GC@OD hydrogels (**b**); Applications of the PB@GC@OD hydrogel in MRSA-infected wound healing (**c**).

This study utilizes oxidized dextran (OD) and GC as the primary scaffold components to construct a GC@OD hydrogel. By integrating soluble Prussian Blue within the hydrogel matrix, we fabricated a PB@GC@OD hydrogel with photothermal antibacterial capability (Figure 1). The OD skeleton, rich in aldehyde groups, reacts with the amine groups on GC via a Schiff-base reaction, forming dynamic and reversible C=N imine bonds. This endows the resulting GC@OD hydrogel with self-healing and injectable properties. Upon incorporation of PB into the GC@OD network, the resultant PB@GC@OD hydrogel rapidly generates heat under NIR laser irradiation, enabling bacterial elimination via localized hyperthermia. Thus, the PB@GC@OD hydrogel retains the self-healing and injectable properties of the GC@OD precursor while gaining potent photothermal bactericidal activity. This dual functionality establishes the hydrogel as a promising candidate for treating infected wounds and accelerating tissue regeneration.

## 2. Materials and Methods

### 2.1. Materials

The following chemical reagents were used as received without any treatment. Sodium periodate, dextran (Dex, Mn = 20,000), Prussian blue soluble (C_6_Fe_2_KN_6_xH_2_O), DMSO-D_6_, and ethylene glycol were bought from Aladdin (Shanghai, China), glycol chitosan (GC) were purchased from Sigma Aldrich (St. Lous, MO, USA, LB agar (Peptone 10.0 g/L, Yeast Extract 5.0 g/L, NaCl 5.0 g/L, Glucose 1.0 g/L, Agar 13.0 g/L, pH = 7.1 ± 0.2) and LB broth (Tryptone 10.0 g/L, Yeast Extract 5.0 g/L, NaCl 10.0 g/L, pH = 7.0 ± 0.2) were obtained from Beijing Luqiao Technology Co., Ltd. (Beijing, China), penicillin, streptomycin, fetal bovine serum, Dulbecco’s Modified Eagle Medium (DMEM), MTT, and Live/Dead staining kit were obtained from Beijing Solarbio Science & Technology Co., Ltd. (Beijing, China).

### 2.2. Synthesis of Oxidized Dextran (OD)

OD was synthesized following a previously reported method [44] with slight modifications. Briefly, 10.0 g of dextran (Dex) was fully dissolved in 200 mL of distilled water, after which 5.2 g of sodium periodate (NaIO_4_), pre-dissolved in distilled water, was added. The reaction was carried out under continuous stirring in the dark at 4 °C for 24 h. Subsequently, 2 mL of ethylene glycol was added, and the mixture was stirred for an additional 2 h to quench unreacted NaIO_4_. Finally, the mixture was transferred into a dialysis bag (MWCO 3.5 kDa) and dialyzed against deionized water for 72 h, followed by lyophilization. The chemical structure of the synthesized OD was characterized by ^1^H nuclear magnetic resonance (^1^H NMR, Bruker AV III HD 500 MHz, Bruker, Karlsruhe, Germany) and Fourier transform infrared (FTIR, Thermo Scientific Nicolet iS50, Thermo Fisher Scientific Inc., Waltham, MA, USA) spectroscopy. The oxidation degree of OD was determined via the oxidation-reduction titration method, following a previously described procedure [45].

### 2.3. Preparation of PB@GC@OD Hydrogels

Stock solutions of Prussian blue (PB) were prepared in phosphate-buffered saline (PBS, pH 7.4) at concentrations of 0.6, 0.45, 0.3, and 0.15 mg/mL. An equal volume of 10% (*w*/*v*) oxidized dextran (OD) solution was added to the PB solution, followed by the addition of an equal volume of 10% (*w*/*v*) glycol chitosan (GC) solution. The mixture was thoroughly vortexed and incubated at room temperature for 5 min to yield PB@GC@OD hydrogels. The final PB concentrations in the hydrogel network were 0.2, 0.15, 0.1, and 0.05 mg/mL, respectively.

To quantitatively determine the PB loading concentration, UV-Vis absorption spectra of standard PB solutions at corresponding concentrations (0.2, 0.15, 0.1, 0.05, and 0.0 mg/mL) were recorded. Based on the comparison of the characteristic absorbance peaks of PB extracted from the PB@GC@OD hydrogels against the standard curve, the final PB concentrations within the hydrogel networks were determined to be 0.2, 0.15, 0.1, and 0.05 mg/mL, respectively. Finally, the gelation time was determined via the vial inversion test.

### 2.4. Photothermal Effect of Hydrogels

The photothermal performance of PB@GC@OD hydrogels was systematically evaluated using an 808 nm near-infrared (NIR) laser system. First, a series of PB@GC@OD hydrogels with varying PB concentrations (0.2, 0.15, 0.1, and 0.05 mg/mL) were prepared. Subsequently, PB@GC@OD hydrogels with the selected PB concentration (0.1 mg/mL) were irradiated with NIR lasers at different power densities (0.5, 1.0, 1.5, and 2.0 W/cm^2^) to identify the optimal laser setting. A power density of 1.0 W/cm^2^ was thereby determined as the optimal parameter. Following this, hydrogels with the four aforementioned PB concentrations were irradiated under the optimized power density (1.0 W/cm^2^) to obtain the corresponding photothermal heating curves. Finally, the photothermal stability of the optimized PB@GC@OD hydrogels (0.1 mg/mL, 1.0 W/cm^2^) was evaluated through three consecutive heating-cooling cycles. Each cycle consisted of 10 min of NIR irradiation followed by passive cooling to room temperature (~25 °C), with temperature data recorded at 30 s intervals using an infrared thermal camera (Hikmicro, H21Pro, Hangzhou, China). All experiments were performed in triplicate.

### 2.5. Scanning Electron Microscopy (SEM) Characterization of Hydrogels

The morphological features of PB_0.1_@GC@OD and GC@OD hydrogels were characterized using SEM (FE-SEM, JEOL-4800, JEOL Ltd., Akishima, Japan). Prior to imaging, hydrogel samples were flash-frozen in liquid nitrogen for 1 h, held at −20 °C for 3 h, and subsequently lyophilized using a freeze dryer (LC-10N-50A, Lichen Bangxi, Shanghai, China). The freeze-dried hydrogels were fractured to reveal the internal morphology, mounted on conductive stages with adhesive carbon tape, sputter-coated with gold, and imaged using SEM.

### 2.6. Adhesive Capacity of the PB_0.1_@GC@OD Hydrogel

The adhesive capacity of the PB_0.1_@GC@OD hydrogel was evaluated. The hydrogel was applied to human finger joints and subjected to continuous shaking for 5 min.

### 2.7. Swelling Measurement of Hydrogels

GC@OD and PB_0.1_@GC@OD hydrogels were prepared and freeze-dried into specimens of uniform geometry. The initial dry weight (*m*_0_) of each sample was recorded before immersion in PBS (pH 7.4).

At predetermined time points, the hydrogels were removed, blotted with filter paper to eliminate excess surface moisture, and weighed (*m*_t_). The swelling ratio was calculated using the following equation. All measurements were conducted in triplicate.

Swelling ratio (%) = (*m*_t_ − *m*_0_)/*m*_0_ × 100%

### 2.8. Water Evaporation Measurement of Hydrogels

Lyophilized GC@OD and PB_0.1_@GC@OD hydrogels were trimmed into similarly shaped and weighted pieces, swollen to equilibrium in PBS, blotted dry, and weighed (*m*_1_). The samples were then placed in a constant temperature and humid environment. At predetermined time intervals, the samples were weighed (*m*_2_). Finally, the hydrogels were completely dried and weighed (*m*_3_). The water evaporation rate was calculated using the following equation. The experiment was repeated three times.

Water evaporation rate = (*m*_1_ − *m*_2_)/(*m*_1_ − *m*_3_) × 100%

### 2.9. Injectability and Self-Healing Capability of Hydrogels

Self-healing ability was qualitatively evaluated by direct observation. Cylindrical hydrogel specimens (2 mm in height and 10 mm in diameter) were bisected, and the resulting cut surfaces were reconnected and incubated at 37 °C. The healing process was visually monitored and documented over time. Quantitative self-healing analysis was performed by oscillatory rheology (Rotational Rheometer, MCR 302, Anton Paar, Graz, Austria). The strain was alternated between low strain (1%, 100 s) and high strain (400%, 100 s) for four cycles near the crossover point of the storage (G′) and loss (G″) moduli. All tests were performed in triplicate.

Injectability was evaluated by loading a mixture comprising 5% (*w*/*v*) OD, 5% (*w*/*v*) GC, and 0.1 μmol/mL PB into a syringe. After gelation inside the syringe, the mixture was extruded through a 22-gauge needle. Additionally, the shear-dependent viscosity of the hydrogel was characterized across shear rates ranging from 1 to 1000 s^−1^.

### 2.10. Rheological Analysis of Hydrogels

Rheological properties of GC@OD and PB_0.1_@GC@OD hydrogels were characterized using an Anton Paar MCR 302 rheometer (Rotational Rheometer, MCR 302, Anton Paar, Graz, Austria). Cylindrical hydrogel samples with a thickness of 2 mm and a diameter of 10 mm were prepared. Amplitude sweep tests were performed at 37 °C with a constant angular frequency of 1 Hz, varying the strain amplitude from 0.1% to 1000%.

Details regarding other experimental procedures, including hemostatic performance of PB_0.1_@GC@OD hydrogels, bacterial culture, in vitro antimicrobial activity assays, cell culture and toxicity assays, hemolysis of the PB_0.1_@GC@OD hydrogels, infected wound healing experiments using PB_0.1_@GC@OD hydrogels, and statistical analysis are provided in the Supplementary Materials.

## 3. Results and Discussion

### 3.1. Preparation and Characterization of PB@GC@OD Hydrogels

Dextran (Dex) is a natural polysaccharide composed of D-glucose units linked by glycosidic bonds. It is biocompatible, non-toxic, and abundant in hydroxyl groups. The vicinal diol groups of the glucose units were oxidized by sodium periodate to generate aldehyde-functionalized oxidized dextran (OD). Subsequently, the OD underwent a Schiff-base reaction with glycol chitosan (GC), which contains abundant amino groups. This reaction yielded a crosslinked hydrogel network through C=N imine bonds (Figure 1a and Figure S1).

**Figure 2:**
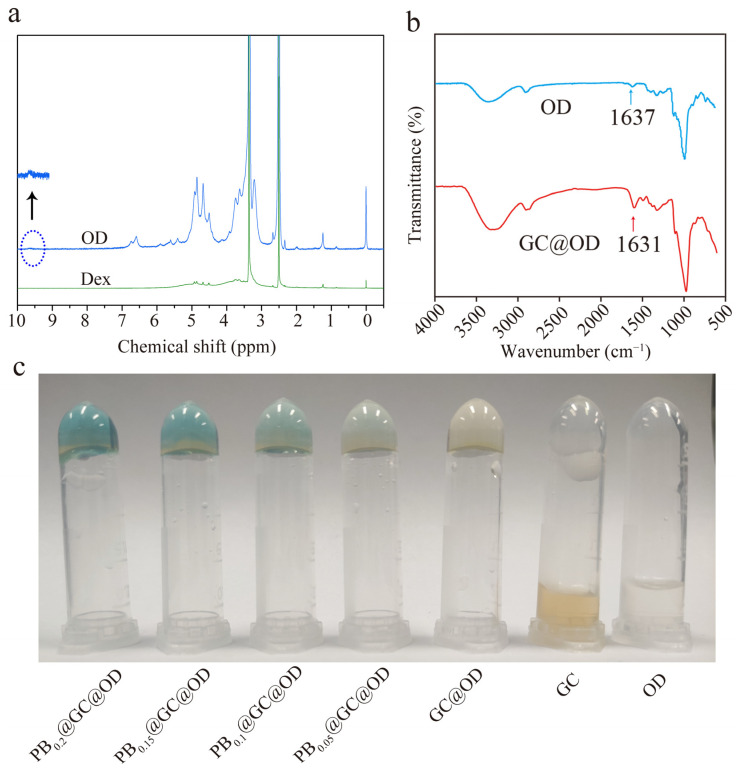
(**a**) ^1^H NMR spectrum of Dex and oxidized dextran (OD) in DMSO-*d*_6_. (**b**) FTIR spectra of GC@OD hydrogel and OD; (**c**) Photographs of GC solution, OD solution, and the PB@GC@OD hydrogels with different PB concentrations.

The chemical structure of OD was initially characterized by ^1^H NMR spectroscopy (Figure 2a). In the ^1^H NMR spectrum of OD, a distinct proton peak was observed at 9.61 ppm, which was absent in native dextran. This new signal was attributed to aldehyde protons generated by the oxidation of vicinal diol groups on the Dex backbone. Furthermore, the FTIR spectrum of OD exhibited a characteristic absorption peak at 1637 cm^−1^ (Figure 2b), assigned to the C=O stretching vibration of aldehyde groups, thereby confirming the successful oxidation of dextran. The oxidation degree of OD was determined to be 35% (Figure S2) via the oxidation-reduction titration method. FTIR analysis of the GC@OD hydrogel revealed a new absorption peak at 1631 cm^−1^. This peak was assigned to the C=N imine bond, indicating that the hydrogel network was successfully formed via Schiff-base crosslinking between GC and OD.

**Figure 3:**
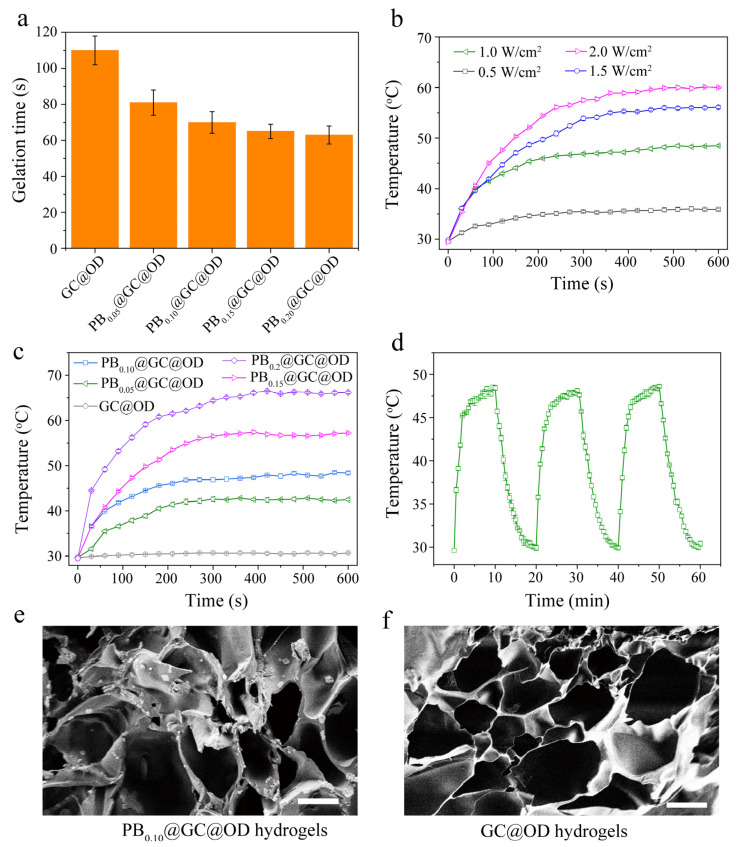
(**a**) Gelation time of PB@GC@OD hydrogels with different PB concentrations (0.2, 0.15, 0.1, 0.05, and 0 mg/mL); (**b**) Photothermal profiles of PB_0.1_@GC@OD hydrogels under NIR irradiation at various power densities; (**c**) Photothermal curves of PB@GC@OD hydrogels with different PB concentrations; (**d**) Photothermal stability of PB_0.1_@GC@OD hydrogel with 3 on/off cycles; (**e**) SEM images of PB_0.10_@GC@OD and (**f**) GC@OD hydrogels.

PB@GC@OD hydrogels with varying PB concentrations (0.2, 0.15, 0.1, and 0.05 mg/mL) (Figure S3) were fabricated by incorporating soluble PB solution into the GC@OD hydrogel matrix (Figure 2c). Macroscopically, the pristine GC@OD hydrogels displayed a pale yellow color, whereas the PB-loaded hydrogels (PB@GC@OD hydrogels) exhibited a characteristic blue hue. Moreover, a higher loading concentration of PB correlated with an intensified blue hue and a shortened gelation time in the PB@GC@OD hydrogel (Figure 3a). Microstructurally, scanning electron microscopy (SEM) revealed that both the pristine and PB@GC@OD hydrogels exhibited a homogeneous three-dimensional interconnected porous architecture (Figure 3e,f). Such a highly porous structure facilitates drug loading and tissue regeneration [46].

### 3.2. Photothermal Performance of PB@GC@OD Hydrogels

PB is a biocompatible and non-toxic inorganic photothermal agent with excellent photothermal conversion efficiency. We first evaluated the photothermal response of PB@GC@OD hydrogels containing 0.1 mg/mL PB under varying power densities (0.5, 1.0, 1.5, and 2.0 W/cm^2^) of 808 nm NIR laser irradiation. As shown in Figure 3b, upon irradiation for 10 min, the hydrogel temperatures reached 36, 48, 56, and 60 °C, respectively. Notably, the temperature approached 50 °C at a power density of 1.0 W/cm^2^; thus, this power density was selected for subsequent experiments.

Subsequently, the photothermal behavior of hydrogels loaded with varying PB concentrations (0.2, 0.15, 0.1, and 0.05 mg/mL) was investigated under continuous 1.0 W/cm^2^ NIR irradiation for 10 min (Figure 3c). The hydrogels reached temperatures of 67, 58, 49, and 42 °C, respectively, demonstrating a distinct concentration-dependent photothermal effect. Notably, hyperthermia above 50 °C induces complete cell necrosis [47], whereas moderate heating (42–47 °C) causes reversible cell damage that can be repaired by heat shock proteins. Importantly, temperatures above 45 °C induce denaturation of bacterial enzymes, disruption of bacterial cell membrane lipids and proteins, and ultimately bacterial death [48]. Therefore, to minimize thermal damage to normal tissue while ensuring effective bacterial eradication, the PB@GC@OD hydrogel with a PB concentration of 0.1 mg/mL was selected as the optimal formulation for subsequent experiments, and we denoted it as the PB_0.1_@GC@OD hydrogel.

The PB_0.1_@GC@OD hydrogel further demonstrated remarkable photothermal stability. As shown in Figure 3d, the temperature variation profiles displayed negligible deviation after three consecutive NIR irradiation cycles, thereby underscoring the hydrogel’s exceptional photothermal durability and its promise as a potent agent for photothermal antibacterial applications.

### 3.3. Rheological Properties, Self-Healing Performance, and Injectability of PB_0.1_@GC@OD Hydrogels

**Figure 4:**
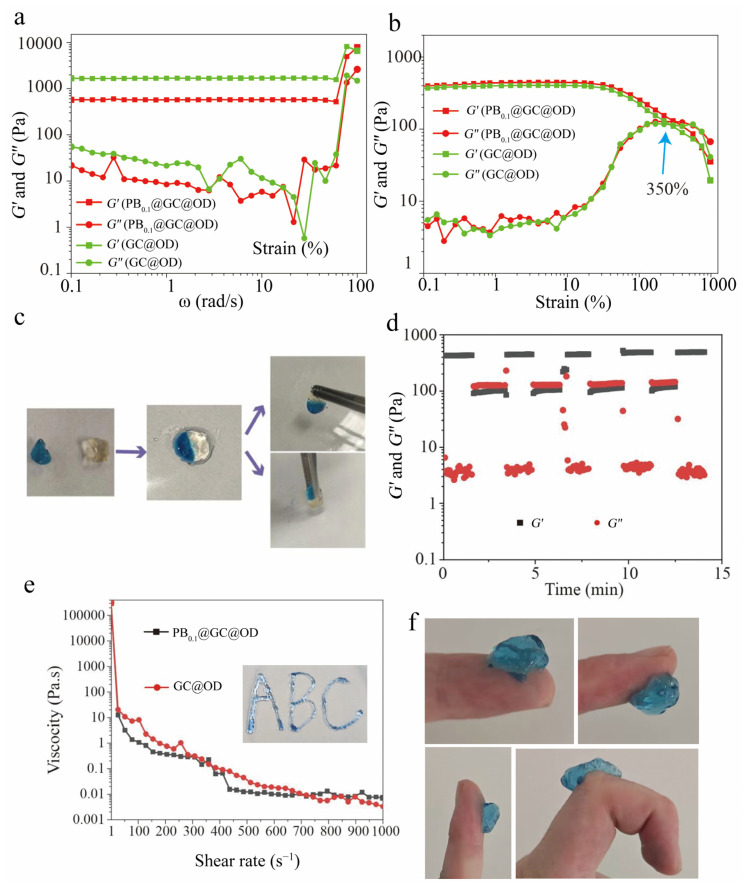
Rheological experiments on PB_0.1_@GC@OD and GC@OD hydrogels. (**a**) Frequency sweep test (γ = 0.5%, ω = 0.1–100 rad/s, T = 37 °C); (**b**) strain amplitude sweep test (γ = 0.1–1000%, ω = 1 Hz, T = 37 °C); (**c**) Images of incision and restoration of PB_0.1_@GC@OD hydrogel; (**d**) Represent the rheological properties of hydrogel alternate step strain switched from 0.1% to 100% (ω = 1 Hz, T = 37 °C, 5 cycles); (**e**) Shear rate-dependent viscosity changes in PB_0.1_@GC@OD hydrogels. Inset: injectability photograph of the PB_0.1_@GC@OD hydrogel; (**f**) Photographs of the adhesive of the PB_0.1_@GC@OD hydrogels on a finger.

The mechanical properties of the hydrogels were characterized using a rotational rheometer. The frequency dependence of the storage modulus (G′) and loss modulus (G″) was measured for both GC@OD and PB_0.1_@GC@OD hydrogels. As shown in Figure 4a, the G′ values were consistently greater than the G″ values across the tested frequency range, confirming that the hydrogels formed stable, elastic-dominant networks. Furthermore, both G′ and G″ exhibited a sharp increase at higher frequencies, a phenomenon characteristic of typical viscoelastic hydrogel behavior. Finally, strain amplitude sweep tests were performed to determine the critical strain limit of the PB_0.1_@GC@OD hydrogels (Figure 4b); the crossover point between the G′ and G″ curves was observed at a strain of 350%. Beyond this critical strain, the G″ value exceeded the G′ value, indicating the disruption of the internal network structure within the PB_0.1_@GC@OD hydrogel and a transition from a gel-like state to a sol-like state.

Self-healing hydrogels present distinct therapeutic potential for treating wounds on dynamic body sites, such as joints and limbs. The macroscopic self-healing capability of the PB_0.1_@GC@OD hydrogel was first demonstrated through a macroscopic cut-and-heal assay. Upon brief contact at 37 °C, the two severed segments fused, making the healing interface virtually indistinguishable. The reconstructed specimen sustained tensile loading without interfacial failure, corroborating effective macroscopic healing (Figure 4c). To quantitatively assess the self-healing kinetics, step-strain amplitude sweeps were conducted, alternating between a low strain and a high strain exceeding the critical limit of 350%. As shown in Figure 4d, upon application of a high strain (400%), the G′ dropped rapidly from 450 Pa to 90 Pa and fell below G″, indicating network breakdown and a gel-to-sol transition. Notably, when the strain was reduced to 1%, G′ rapidly regained its initial value (~450 Pa) and exceeded G″, demonstrating an instantaneous sol-to-gel transition and restoration of the network structure. This rapid self-healing ability is attributed to dynamic Schiff-base linkages (C=N bonds) within the hydrogel network. Upon structural damage, the reversible imine bonds dissociate and re-form between the amino groups of GC and the aldehyde groups of OD, thereby facilitating autonomous reconstruction of the cross-linked network.

The injectability of the PB_0.1_@GC@OD hydrogel, a property intrinsically linked to its self-healing capability, was further evaluated. PB_0.1_@GC@OD hydrogel was successfully extruded through a 20-gauge needle to form the letters “ABC” without clogging (inset of Figure 4e). This macroscopic behavior is governed by the hydrogel’s rheological properties. As shown in Figure 4d, the viscosity of the hydrogel decreased sharply with increasing shear rate, demonstrating characteristic shear-thinning behavior. This property facilitates a reversible transition from a gel-like to a sol-like state under shear stress, thereby enabling injection through narrow-gauge needles. PB_0.1_@GC@OD hydrogel firmly adhered to the skin surface of a finger and remained attached even during repeated bending and movement, without detachment or fragmentation (Figure 4f). Such favorable tissue adhesion allows the hydrogel to conform closely to irregular wound surfaces and remain in place during daily activities, which is critical for maintaining a stable moist healing environment and preventing secondary infection. Collectively, these superior injectability and self-healing attributes suggest that the PB_0.1_@GC@OD hydrogel is a promising candidate for minimally invasive therapies and the management of irregular wounds.

### 3.4. Swelling Behavior and Moisturizing Capacity of PB_0.1_@GC@OD Hydrogels

**Figure 5:**
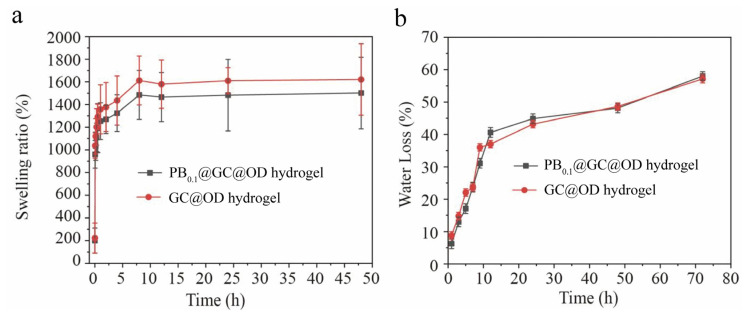
Swelling and water retention of hydrogels. (**a**) Swelling rate of PB_0.1_@GC@OD hydrogel and GC@OD hydrogel. (**b**) Water evaporation rate of PB_0.1_@GC@OD hydrogel and GC@OD hydrogel.

The PB_0.1_@GC@OD hydrogel exhibited exceptional water absorption and retention properties, critical for maintaining a moist wound environment. Leveraging its porous network structure, the hydrogel rapidly absorbed water upon contact with water, as depicted in Figure 5a. The absorption rate diminished as swelling equilibrium was approached. The system achieved an equilibrium swelling ratio of 1500–1700%, which is comparable to that of the GC@OD hydrogel, indicating that PB incorporation did not compromise swelling performance.

The hydrogel’s moisture retention capacity is illustrated in Figure 5b. Initially, water evaporation was rapid. However, the rate decreased sharply after 10 h, with 42% of the initial moisture content retained after 72 h. This exceptional retention is attributed to capillary forces and hydrogen bonding within the dense three-dimensional network, which effectively retain water within the matrix. These properties facilitate prolonged wound hydration and protection against secondary damage, thereby accelerating the healing of infected wounds.

### 3.5. Cytotoxicity and Hemocompatibility of PB_0.1_@GC@OD Hydrogels

**Figure 6:**
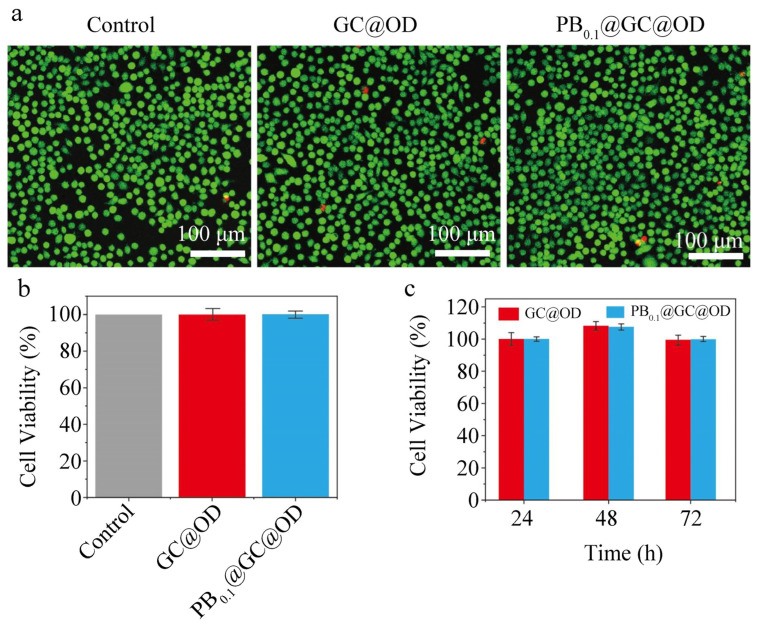
Cytocompatibility of hydrogels. (**a**) Live & Dead staining images of L929 cells treated with PBS (control), leaching solutions of GC@OD hydrogels and PB_0.1_@GC@OD hydrogels for 24 h. Scale bar: 100 μm. (**b**) Cell viability of L929 cells co-cultured with PBS, leaching solutions of GC@OD hydrogels and PB_0.1_@GC@OD hydrogels for 24 h. (**c**) Cell viability of L929 cells co-cultured with leaching solutions of GC@OD hydrogels and PB_0.1_@GC@OD hydrogels for 24, 48 and 72 h.

Live/dead staining assays (Figure 6a) revealed that cell viability in groups treated with hydrogel extracts after 24 h of incubation was comparable to that of the control group. These findings were corroborated by MTT assays, which showed cell viability exceeding 95% across all experimental groups following 24 h of exposure (Figure 6b). Moreover, cell viability remained comparable to the control group after 48 and 72 h of incubation with hydrogel extracts, suggesting that both GC@OD and PB_0.1_@GC@OD hydrogels exhibit no cytotoxicity toward L929 fibroblasts (Figure 6c). Notably, the PB_0.1_@GC@OD hydrogel displayed no significant cytotoxicity, thereby validating the biocompatibility of this optimal formulation.

**Figure 7:**
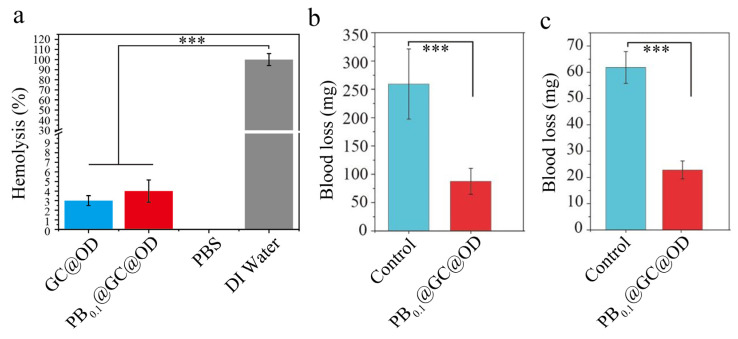
(**a**) Hemolysis rate of PB_0.1_@GC@OD hydrogel; (**b**) In vivo hemostatic performance of PB_0.1_@GC@OD hydrogel in the hemorrhaging liver mouse model and (**c**) in vivo hemostatic performance of PB_0.1_@GC@OD hydrogel in the hemorrhaging tails mouse model. *** *p* < 0.001.

Moreover, the hemolytic activity of the hydrogels was evaluated using red blood cells. As shown in Figure 7a and Figure S4, the hemolysis rates of both GC@OD and PB_0.1_@GC@OD hydrogels were below the 5% safety threshold established for biomaterials, indicating negligible hemolytic potential. The absence of a significant difference in hemolysis rates between the two hydrogels (*p* < 0.001) provides strong evidence that PB incorporation does not enhance hemolytic activity. Consequently, these results establish the PB_0.1_@GC@OD hydrogel as a safe, non-toxic, and biocompatible wound dressing with potential for clinical translation, thereby addressing the critical need for effective antibacterial wound management.

### 3.6. Hemostatic Performance of PB_0.1_@GC@OD Hydrogels

The hemostatic efficacy of the PB_0.1_@GC@OD hydrogel was rigorously assessed in a mouse liver injury model. As shown in Figure 7b, the blood loss in liver wounds treated with the PB_0.1_@GC@OD hydrogel (79.5 ± 22.8 mg) was significantly reduced compared to the control group (259.4 ± 87.6 mg, *p* < 0.001). These results were corroborated in a tail amputation hemorrhage model (Figure 7c), in which the blood loss in the hydrogel-treated group (22.5 ± 3.4 mg) was substantially lower than that in the control group (61.8 ± 6.6 mg; *p* < 0.001). Collectively, these results demonstrate that the PB_0.1_@GC@OD hydrogel exhibits superior hemostatic performance across diverse hemorrhage models.

The exceptional hemostatic efficacy of the PB_0.1_@GC@OD hydrogel is attributed to its dual-functional mechanism. First, the hydrogel’s stable porous architecture promotes platelet activation and red blood cell entrapment, thereby accelerating the coagulation cascade. Second, the aldehyde groups within the hydrogel matrix form covalent bonds with amino groups on tissue surfaces. Through synergistic physical adhesion and interfacial chemistry, including hydrogen bonding and cation-π interactions, the hydrogel forms a robust adhesive seal at the hemorrhagic site [49]. This distinctive integration of structural and chemical properties enables the PB_0.1_@GC@OD hydrogel to rapidly adhere to wound sites and establish an effective barrier against blood loss.

### 3.7. Photothermal Antimicrobial Activity of PB_0.1_@GC@OD Hydrogels

**Figure 8:**
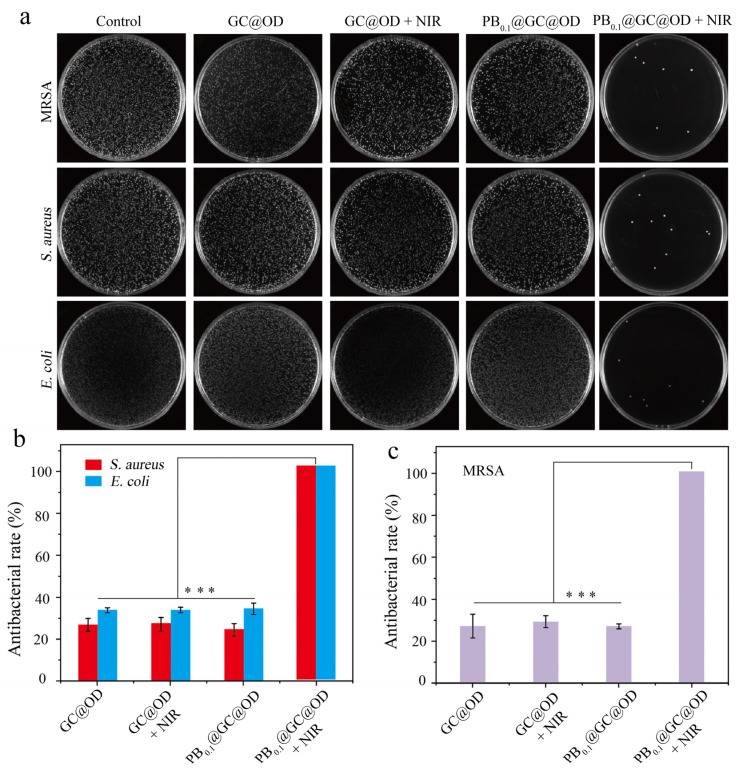
Antimicrobial properties of hydrogels. (**a**) Photographs of the agar plates of *S. aureus*, *E. coli* and *MRSA* colonies after incubation with PBS, GC@OD, PB_0.1_@GC@OD hydrogels and hydrogel after infrared light irradiation. (**b**) The corresponding statistical antibacterial results of *S. aureus* and *E. coli* according to (**a**). (**c**) The corresponding statistical antibacterial results of *MRSA* according to (**a**). *** *p* < 0.001.

As shown in Figure 8, the GC@OD hydrogel exhibited moderate antibacterial activity against *S*. *aureus*, *E*. *coli*, and MRSA, attributable to the protonation of amino groups (-NH_3_^+^). In contrast to the GC@OD hydrogel, the PB_0.1_@GC@OD hydrogel without NIR irradiation exhibited no significant improvement in antimicrobial activity against all three strains, indicating that PB itself lacks intrinsic bactericidal properties. Conversely, under 808 nm NIR laser irradiation (1.0 W/cm^2^, 5 min), the PB_0.1_@GC@OD hydrogel eradicated 99.9% of the bacteria across all three strains. The exceptional antibacterial performance is attributed to the efficient photothermal conversion of PB: upon NIR irradiation, the PB_0.1_@GC@OD hydrogel rapidly reaches temperatures exceeding 45 °C, inducing enzyme denaturation and membrane disruption, which collectively lead to bacterial death.

### 3.8. Wound-Healing Efficacy of PB_0.1_@GC@OD Hydrogel in Infected Wounds

**Figure 9:**
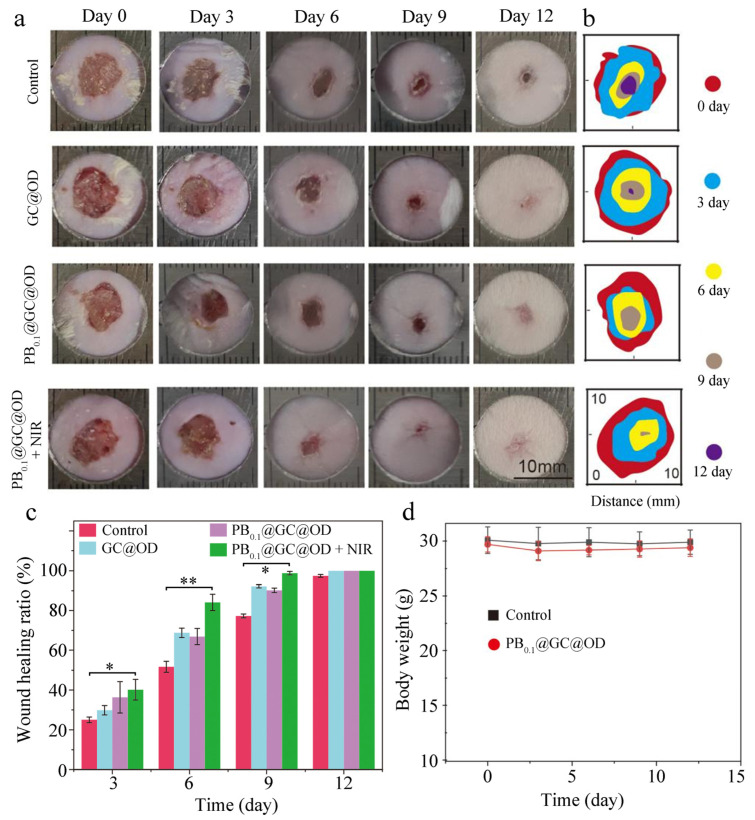
In vivo MRSA-infected wound healing. (**a**) Images of the wound healing process within 14 days after different treatments (scale bar: 10 mm). (**b**) Schematic images of wound contraction during 14 days for different treatments. (**c**) Quantitative analysis of the healing rate of the wound according to (**a**). * *p* < 0.05, ** *p* < 0.01. (**d**) Relative body weight changes in mice within 14 days after different treatments.

As shown in Figure 9a, wound healing progression was monitored on days 0, 3, 6, 9, and 12 post-treatment. By day 6, the wound healing rates in the PB_0.1_@GC@OD + NIR, PB_0.1_@GC@OD, and GC@OD groups were 82%, 62%, and 63%, respectively, whereas the control group reached only 51%. Notably, the PB_0.1_@GC@OD + NIR group demonstrated significantly accelerated wound healing compared to the PB_0.1_@GC@OD and GC@OD groups; this enhancement is attributed to the synergy between PB-mediated photothermal effects and the hydrogel’s intrinsic antibacterial activity driven by ammonium ion generation. By day 9, wounds in the PB_0.1_@GC@OD + NIR group achieved complete closure, whereas the rates for the PB_0.1_@GC@OD and GC@OD groups were 95% and 90%, respectively, in contrast to 70% for the control group. These results confirmed the PB_0.1_@GC@OD hydrogel as a promising dual-mode therapeutic platform for infected wound management, with NIR irradiation maximizing its therapeutic efficacy.

**Figure 10:**
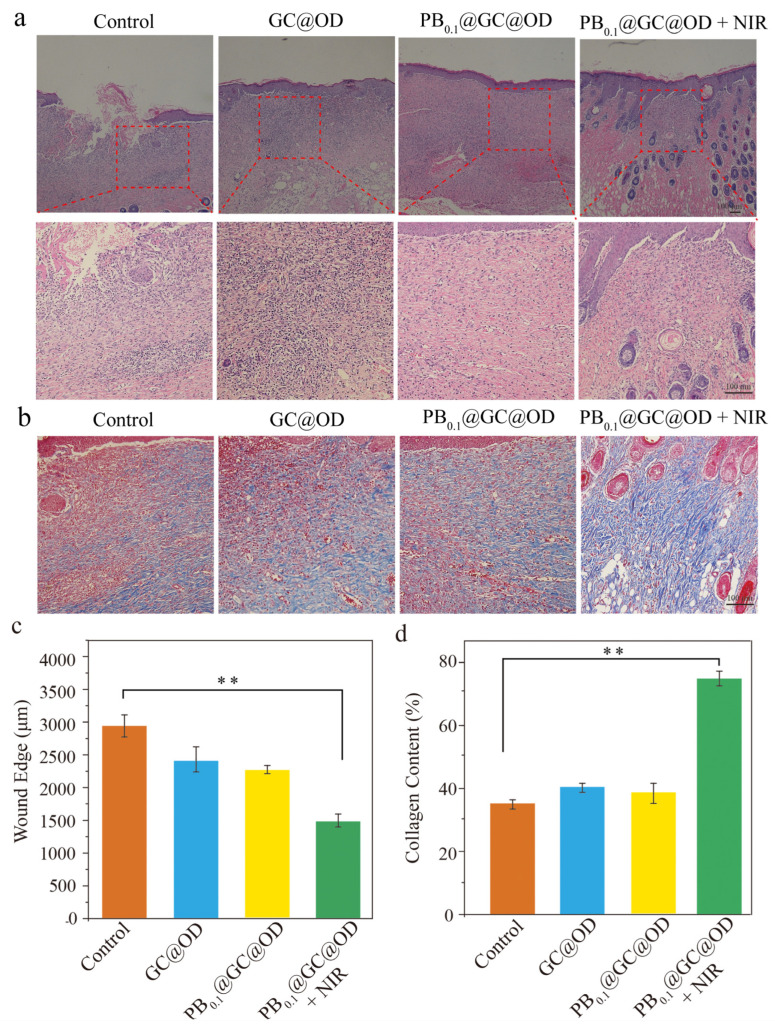
Staining of wound sections after 14 days. (**a**) Images of H&E-stained (scale bar: 100 μm) and (**c**) Masson-stained (scale bar: 100 μm) wound sections on the 12th day. (**b**) Quantitative analysis of the wound edge based on (**a**). (**d**) Quantitative analysis of collagen content deposited based on (**c**). ** *p* < 0.01.

More importantly, H&E staining revealed incomplete tissue regeneration in the control group by day 12 post-treatment, while samples from the PB_0.1_@GC@OD + NIR, PB_0.1_@GC@OD, and GC@OD groups exhibited complete re-epithelialization, indicative of full wound closure. Notably, the PB_0.1_@GC@OD + NIR group displayed a significantly smaller wound area and diminished inflammatory cell infiltration compared to the control group (Figure 10a,c), consistent with the macroscopic observations. These results underscore the superior therapeutic efficacy in promoting wound healing of the PB_0.1_@GC@OD + NIR treatment.

To evaluate collagen deposition, Masson’s trichrome staining was subsequently conducted. As illustrated in Figure 10b,d, collagen deposition, characterized by intense blue staining, was significantly greater in the PB_0.1_@GC@OD + NIR group compared to all other groups. Quantitative analysis indicated that the area of collagen deposition accounted for 76% in the PB_0.1_@GC@OD + NIR group, whereas it constituted only 35% in the control group. Collectively, these results demonstrate that PB-mediated photothermal conversion under NIR irradiation (808 nm, 1.0 W/cm^2^) synergistically augments the hydrogel’s intrinsic antibacterial efficacy, thereby alleviating bacterial burden and inflammation and promoting collagen deposition via the preservation of an optimal moist microenvironment. This dual-mode therapeutic strategy validates the PB_0.1_@GC@OD hydrogel as a robust platform for treating MRSA-infected wounds, wherein NIR irradiation activates its comprehensive therapeutic potential.

### 3.9. In Vivo Safety Evaluation of PB_0.1_@GC@OD Hydrogels

**Figure 11:**
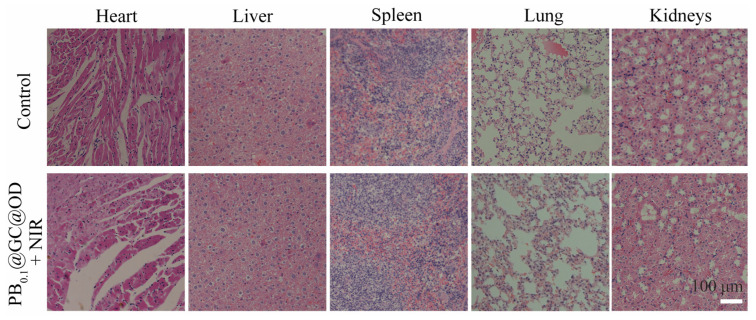
H&E-stained tissue images of the major organs in normal uninfected mice (control group) and the MRSA-infected mice treated with PB_0.1_@GC@OD hydrogel (scale bar = 100 μm).

As shown in Figure 11, histological examination revealed no evident signs of inflammation or tissue damage in the major organs (heart, liver, spleen, lungs, and kidneys) of mice in either the control or PB_0.1_@GC@OD + NIR group, suggesting the biocompatibility of the hydrogel for potential long-term management of infected wounds. Figure 9d illustrates that the body weights of mice in the hydrogel-treated group remained stable throughout the 12-day experimental period, thereby confirming the absence of systemic toxicity and adverse effects on the general health of the subjects.

## 4. Conclusions

In conclusion, a GC@OD hydrogel was fabricated through a Schiff-base reaction between aldehyde groups in OD and amino groups in GC, forming reversible C=N imine bonds. Subsequently, PB was incorporated into the GC@OD hydrogel, yielding a PB_0.1_@GC@OD hydrogel exhibiting photothermal antimicrobial activity. This multifunctional hydrogel integrates self-healing, injectability, and efficient photothermal conversion, facilitating rapid induction of local hyperthermia under 808 nm near-infrared irradiation for bacterial eradication. In vitro assays demonstrated potent bactericidal effects against *S. aureus*, *E. coli*, and MRSA under near-infrared irradiation. Furthermore, in vivo studies validated its remarkable efficacy in accelerating MRSA-infected wound healing by maintaining a moist microenvironment and photothermally abating pathogens, all while ensuring biocompatibility and long-term safety. Overall, the developed PB_0.1_@GC@OD hydrogel represents a promising wound dressing that integrates photothermal sterilization, self-healing capability, hemocompatibility, hemostatic performance, and biocompatibility. This combination of properties underscores its potential as an effective and versatile platform for treating bacteria-infected wounds, thereby offering a robust strategy to simultaneously address microbial elimination and tissue regeneration.

## Data Availability

The original contributions presented in this study are included in the article/Supplementary Materials. Further inquiries can be directed to the corresponding authors.

## Supplementary Materials

The following supporting information can be downloaded at: https://www.mdpi.com/article/10.3390/polym18141688/s1.
