# Supplementary material for: Multifunctional Prussian-Blue-Based Hydrogel for Photothermal Antibacterial and Infected Wound Regeneration
Source: Polymers (Basel). 2026 Jul 9;18(14):1688. doi: 10.3390/polym18141688 (PMC13416061; doi:10.3390/polym18141688)
Supplement: Supplementary file 1 [file polymers-18-01688-s001.zip › polymers-4322696-supplementary.pdf]

Supplementary Materials

Multifunctional Prussian-Blue-Based Hydrogel for Photothermal Antibacterial and Infected Wound Regeneration

Shiqi Gao [1,2,†], Minzhen Liu [1,2,†], Jiteng Sun [1,3,†], Zhicheng Su [1,2], Ziyun Liao [1,2], Peiyu Li [1,3], Yunqi Jiang [3], Can Fu [1,2,4,*] and Guangyu Pan [1,2,3,5,*]

[1] Guangxi Key Laboratory of Diabetic Systems Medicine, Guilin Medical University, Guilin 541199, China; shiqi@stu.glmc.edu.cn (S.G.); 19175931257@163.com (M.L.); 13978423864@stu.glmc.edu.cn (Z.S.); perry1271522559@gmail.com (P.L.)

[2] School of Pharmacy, Guilin Medical University, Guilin 541199, China

[3] School of Artificial Intelligent Medicine, Guilin Medical University, Guilin 541199, China

[4] School of Basic Medical Sciences, Guilin Medical University, Guilin 541199, China

[5] Key Laboratory of Biochemistry and Molecular Biology (Guilin Medical University), Education Department of Guangxi Zhuang Autonomous Region, Guilin 541199, China

* Correspondence: 17877358308@163.com (C.F.); guangyupan@glmc.edu.cn (G.P.)

† These authors contributed equally to this work.

2.10 Hemostatic Performance of Hydrogels

All animal experiments performed in this study were conducted in strict accordance with the National Research Council's Guide for the Care and Use of Laboratory Animals, and were approved by the Animal Experimentation Ethics Committee of Guilin Medical University (approval number: GLMC202103275, date of approval 9 March 2021). The experiments were conducted under the supervision of the Committee, in strict adherence to the approved experimental protocol and ethical guidelines. The hemostatic efficacy of the $PB_{0.1}$@GC@OD hydrogel was evaluated using a mouse liver hemorrhage model and a mouse tail amputation model.

For the mouse liver hemorrhage model, KM mice (female, 30-35 g) were anesthetized and immobilized on a surgical board. A midline abdominal incision (1-2 cm in length) was made to expose the liver. Subsequently, a standardized bleeding wound (approximately 5 mm in diameter) was induced on the liver surface. Immediately after inducing hemorrhage, 100 μL of the $PB_{0.1}$@GC@OD hydrogel was directly applied to the bleeding site. After 3 minutes, the exuded blood was absorbed using clean, dry filter paper and quantified by weighing. Control mice, subjected to an identical liver injury, received no treatment. All outcomes were photographically documented. Each experimental condition was tested in triplicate.

In the tail-amputation model, following anesthesia, the mouse tail was transected 3 cm from the distal tip. The wound was immediately treated with 30 μL of the $PB_{0.1}$@GC@OD hydrogel. Blood exudate was absorbed onto pre-weighed filter

paper for 3 minutes, and the total blood loss was quantified based on the gravimetric difference. Mice in the control group received no therapeutic intervention.

2.11 Culture of bacteria

Staphylococcus aureus (*S. aureus*), *Escherichia coli* (*E. coli*), and MRSA were streaked onto LB agar plates and incubated at 37°C for 12 h. Single colonies were picked and inoculated into 8 mL of LB broth, followed by overnight incubation at 37 °C with agitation at 180 rpm. Subsequently, 100 μL of the overnight seed culture was transferred into 8 mL of fresh LB broth and incubated for an additional 2 h. Bacterial cells were harvested via centrifugation, washed twice with sterile physiological saline, and resuspended in the same buffer to an optical density at 600 nm (OD600) of 0.5, corresponding to approximately 1×10^8 CFU/mL. The resulting bacterial suspension was maintained at 4°C until immediate use.

2.12 In vitro antimicrobial activity assay

The in vitro antibacterial activity of the $PB_{0.1}$@GC@OD hydrogel was evaluated using the plate counting method against *E. coli*, *S. aureus*, and MRSA. Hydrogel samples were prepared as cylinders (10 mm diameter, 2 mm height) and placed in 12-well plates, followed by sterilization under UV irradiation. The bacterial suspension (1×10^8 CFU/mL) was incubated with the hydrogels to ensure complete contact. The samples were then divided into two groups: the NIR group, which was exposed to 808 nm near-infrared light for 10 min, and the dark group, which was kept in the dark for the same duration. Subsequently, both groups were incubated at 37 °C for 2 h. A control group without hydrogel was also prepared. After co-incubation, the bacterial suspensions were serially diluted, plated on LB agar, and incubated at 37 °C for 24 h. All experiments were performed in triplicate, and the antibacterial rates were calculated using the following formula:

Bactericidal rate (%) = $(A_1 - A_2)/A_1 \times 100\%$

A_1 and A_2 represent the colony-forming units (CFUs) of the blank control and experimental groups, respectively.

2.13 Cell culture and toxicity assays

Mouse fibroblast cells (L929), obtained from the School of Artificial Intelligence Medicine at Guilin Medical University, were cultured in DMEM supplemented with streptomycin (100 μg/mL), penicillin (100 μg/mL), and fetal bovine serum (10%). The cytotoxicity of the $PB_{0.1}$@GC@OD hydrogels was evaluated using the MTT assay and live/dead cell staining.

For extract preparation, freeze-dried hydrogel samples were incubated in culture medium at 37°C for 24 h. L929 cells in the logarithmic growth phase were trypsinized and seeded into 96-well plates (5×10^4 cells per well). After 24 h of pre-culture, the medium was replaced with medium containing the hydrogel extract, and the cells were incubated for periods of 24, 48, and 72 h. Control cells were maintained in fresh culture medium. All experiments were conducted in triplicate.

For Live/Dead staining, cells were stained with a commercial kit and visualized under a fluorescence microscope (TI2-N ND-I, Nikon, Japan) after 24 h. For the MTT assay, following co-culture periods of 1, 2, and 3 days, 10 μL of MTT solution (5 mg/mL) was added to each well, followed by a 4-h incubation. Subsequently, 150 μL of dimethyl sulfoxide (DMSO) was added to dissolve the formazan crystals. The absorbance was measured at 490 nm using a microplate reader, and the relative cell viability was calculated using the following formula:

$$\text{Relative cell survival rate (\%)} = (C_1 - C_0) / (C_2 - C_0) \times 100\%$$

C_0, C_1, and C_2 denote the mean absorbance values of the blank, experimental, and control groups, respectively. All experiments were conducted in triplicate.

2.14 Hemolysis of hydrogels

All animal experiments performed in this study were conducted in strict accordance with the National Research Council's Guide for the Care and Use of

Laboratory Animals, and were approved by the Animal Experimentation Ethics Committee of Guilin Medical University (approval number: GLMC202103275, date of approval 9 March 2021). The experiments were conducted under the supervision of the Committee, in strict adherence to the approved experimental protocol and ethical guidelines.

The hemolytic activity of the $PB_{0.1}$@GC@OD hydrogel was evaluated using a previously described method. Briefly, KM mouse were anesthetized, and fresh whole blood was collected by retro-orbital bleeding using heparinized capillary tubes. The collected blood (1.0 mL) was centrifuged at 1000 rpm for 10 min. Then the supernatant was discarded, and 10 mL of PBS was added. This centrifugation and washing cycle was repeated three times until the supernatant appeared colorless, indicating the complete removal of plasma components. The resulting erythrocyte pellet was resuspended in 1.0 mL of PBS to yield a working red blood cell suspension with a concentration of approximately 5×10^6 cells/mL.

Freeze-dried hydrogel samples were incubated in PBS at 37 °C for 24 h to prepare hydrogel extracts, which were then sterile-filtered through a 0.22 μm membrane. 500 μL aliquots of the working red blood cell suspension were mixed with an equivalent volume of the hydrogel extract. Distilled water and PBS served as the positive and negative controls, respectively. All experiments were performed in triplicate. Following incubation at 37 °C for 60 min, all samples were centrifuged at 1000 rpm for 10 min.

After centrifugation, images of the EP tubes were acquired to visually assess hemolysis. Subsequently, 100 μL of the supernatant was aliquoted into a 96-well plate, and the absorbance was recorded at 540 nm using a microplate reader. The relative hemolysis rate was calculated according to the following equation:

Relative hemolysis rate (%) = $(B_1 - B_0)/ (B_2 - B_0) \times 100\%$

Where B_0, B_1, and B_2 denote the mean absorbance of the negative control, the test samples, and the positive control, respectively. Each group was tested in triplicate.

2.15 Infection-promoting wound healing experiment with $PB_{0.1}$@GC@OD hydrogel

All animal experiments performed in this study were conducted in strict accordance with the National Research Council's Guide for the Care and Use of Laboratory Animals, and were approved by the Animal Experimentation Ethics Committee of Guilin Medical University (approval number: GLMC202103275, date of approval 9 March 2021). The experiments were conducted under the supervision of the Committee, in strict adherence to the approved experimental protocol and ethical guidelines.

The wound-healing efficacy of the GC@OD hydrogel was evaluated using an MRSA-infected wound model in female Kunming mice (30 – 40 g). The Kunming mice purchased from the Animal Experiment Center of Guilin Medical University were maintained on a standard diet and housed in separate cages within an SPF-grade sterile animal facility.

Sixteen female Kunming mice were randomly allocated into four distinct experimental groups: Control, GC@OD, $PB_{0.1}$@GC@OD, and $PB_{0.1}$@GC@OD + NIR. Prior to procedural initiation, the mice were anesthetized by intraperitoneal injection of 0.94% sodium pentobarbital. Dorsal fur was shaved and chemically depilated, followed by disinfection of the exposed skin with 75% ethanol. An 8-mm-diameter full-thickness excisional wound was induced on the dorsal surface utilizing a sterile stainless steel biopsy punch and surgical scissors. Subsequently, the wound bed was inoculated with 20 μL of MRSA suspension (1×10^8CFU/mL) to establish the infection model.

In the treatment group, the hydrogel was topically applied to the wound every 3 days, while the control group received a gauze dressing on the same schedule. Before each treatment, wound dimensions were quantitatively assessed using digital calipers and recorded via digital imaging. The wound healing rate (%) was calculated using the following formula:

Wound healing rate (%) = $(H_1 - H_0)/B_0 \times 100\%$

where H_0 and H_1 denote the initial wound area and the wound area at a specific time point post-treatment, respectively.

On day 12 post-treatment initiation, the mice in each group were photographed and then euthanized. Skin tissue samples from the wound sites and major organs, including the heart, liver, spleen, lungs, and kidneys, were collected and fixed in 4% paraformaldehyde solution. Tissue sections were subsequently prepared, stained with hematoxylin and eosin (H&E) and Masson's trichrome, and then imaged, and subsequently imaged using light microscopy.

2.16 Statistical analysis

Data were presented as the mean ± standard deviation. Differences between two groups were analyzed using the t-test, while comparisons among multiple groups were analyzed by one-way analysis of variance (ANOVA). Statistical significance was set at $P < 0.05$, and $*P < 0.01$ was defined as highly significant.

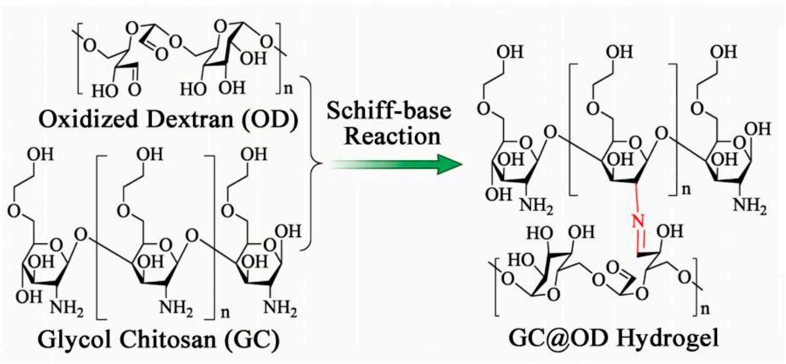

Figure S1. Preparation of the GC@OD hydrogel.

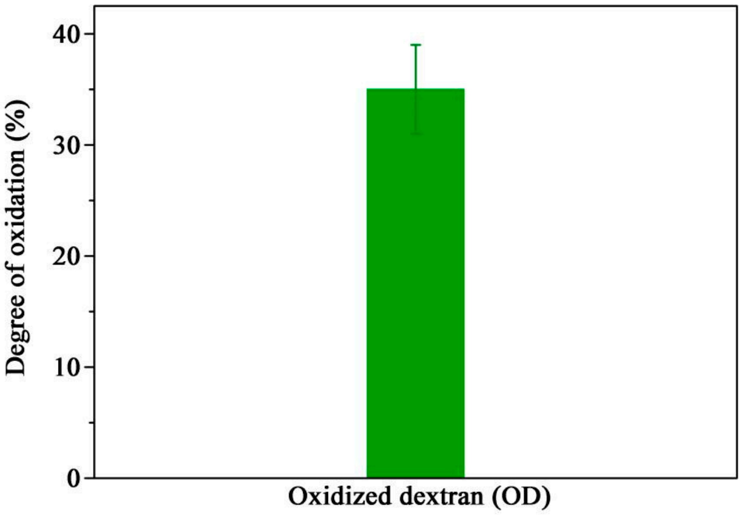

Figure S2. The oxidation degree of Oxidized Dextran (OD) (n = 3).

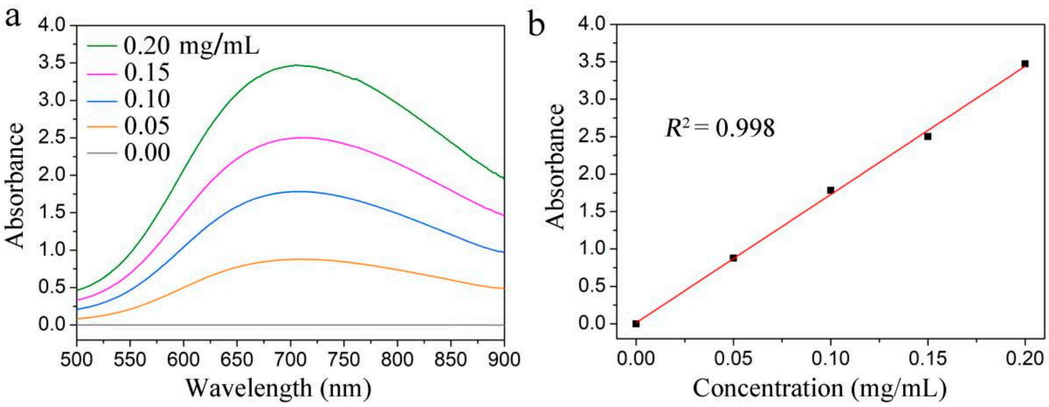

Figure S3. (a) UV-Vis absorbance of PB at concentrations of 0.2, 0.15, 0.1, 0.05, and 0.0 mg/mL. (b) Linear correlation between maximum absorbance and concentration shown in Figure S3a.

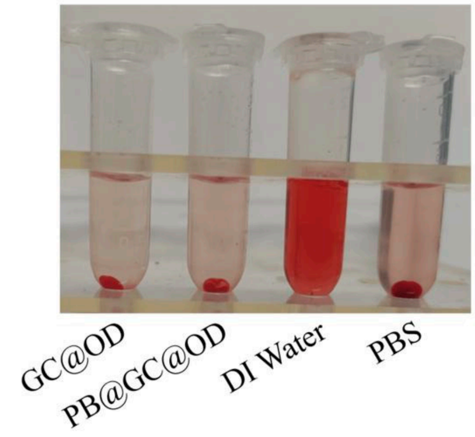

Figure S4. Photographs of hemolysis test samples.
